# Halothane potentiates the alcohol-adduct induced TNF-alpha release in heart endothelial cells

**DOI:** 10.1186/1471-2253-5-3

**Published:** 2005-04-12

**Authors:** Geoffrey M Thiele, Gary E Hill, Jacqueline A Pavlik, Thomas L Freeman, Dean J Tuma, Michael J Duryee, Lynell W Klassen

**Affiliations:** 1 University of Nebraska Medical Center, Department of Internal Medicine, 988090 Nebraska Medical Center, Omaha, NE, 68198-3025, USA; 2 Veterans Administration Alcohol Research Center, Omaha Veterans Administration Medical Center, 4101 Woolworth Avenue, Omaha, NE, 68105, USA; 3 University of Nebraska Medical Center, Department of Pathology and Microbiology, 986495 Nebraska Medical Center, Omaha, NE, 68198-6495, USA; 4 UT South western, Department of Anesthesiology and Pain Management, 5323 Harry Hines Blvd., Dallas, TX, 75390-9072, USA

## Abstract

**Background:**

The possibility exists for major complications to occur when individuals are intoxicated with alcohol prior to anesthetization. Halothane is an anesthetic that can be metabolized by the liver into a highly reactive product, trifluoroacetyl chloride, which reacts with endogenous proteins to form a trifluoroacetyl-adduct (TFA-adduct). The MAA-adduct which is formed by acetaldehyde (AA) and malondialdehyde reacting with endogenous proteins, has been found in both patients and animals chronically consuming alcohol. These TFA and MAA-adducts have been shown to cause the release of inflammatory products by various cell types. If both adducts share a similar mechanism of cell activation, receiving halothane anesthesia while intoxicated with alcohol could exacerbate the inflammatory response and lead to cardiovascular injury.

**Methods:**

We have recently demonstrated that the MAA-adduct induces tumor necrosis factor-α (TNF-α) release by heart endothelial cells (HECs). In this study, pair and alcohol-fed rats were randomized to receive halothane pretreatments intra peritoneal. Following the pretreatments, the intact heart was removed, HECs were isolated and stimulated with unmodified bovine serum albumin (Alb), MAA-modified Alb (MAA-Alb), Hexyl-MAA, or lipopolysaccharide (LPS), and supernatant concentrations of TNF-α were measured by ELISA.

**Results:**

Halothane pre-treated rat HECs released significantly greater TNF-α concentration following MAA-adduct and LPS stimulation than the non-halothane pre-treated in both pair and alcohol-fed rats, but was significantly greater in the alcohol-fed rats.

**Conclusion:**

These results demonstrate that halothane and MAA-adduct pre-treatment increases the inflammatory response (TNF-α release). Also, these results suggest that halothane exposure may increase the risk of alcohol-induced heart injury, since halothane pre-treatment potentiates the HEC TNF-α release measured following both MAA-Alb and LPS stimulation.

## Background

Anesthetics like halothane are rarely used in most nations except in developing countries, which still widely use this method of anesthesia [1]. Also on the rise is alcohol consumption in developing countries [2]. Patients consuming alcohol who are anesthetized with halothane could potentially have inadequate metabolism or adduct formation leading to problems such as cardiovascular disease or liver injury. Hepatic metabolism of halothane and ingested ethanol (ethyl alcohol, alcohol) yields the highly reactive metabolites: trifluoroacetyl chloride (TFA) from halothane and acetaldehyde (AA) and malondialdehyde (MDA) from the oxidation of ethanol [3,4]. MDA and AA react together with endogenous proteins (most likely the ε-amino group of lysine residues) to form distinctive new adducted proteins [3,4]. The adduct formed by the combination of MDA and AA has been termed the MAA-adduct by Tuma *et al *[4] and has been detected in humans and rats chronically consuming ethanol [5,6]. In fact, Slatter *et al *[7] have recently confirmed that MDA, AA, and lysine react to form a dihydropyridine derivative structurally identical to the MAA-adduct. Similarly, trifluoroacetyl chloride (TFA) will react with amine groups to form a distinctive protein termed the TFA-adduct [8].

Recent reports by our laboratory have demonstrated that the MAA-adduct will induce the release of the proinflammatory cytokine tumor necrosis factor-alpha (TNF-α) in a purified rat heart endothelial cell culture (HEC) [9]. Trudell *et al *[3] reported data suggesting that the TFA-adduct may cause cell injury by inducing a similar inflammatory response. Importantly, it has been demonstrated that the TFA-adduct is present in heart tissue obtained from halothane pre-treated rats [10,11]. If both adducts share a similar mechanism of cell activation, receiving halothane anesthesia while intoxicated with alcohol could exacerbate the inflammatory response. Also of interest is that both halothane and ethanol are metabolized through cytochrome P450 2E1 (CYP2E1) [12], possible providing a shared mechanism. In support of this is data suggesting that acetaldehyde effects ventricular myocyte contraction through mechanisms related to CYP oxidase and lipid peroxidation [13]. This could help explain how ethanol consumption and halothane anesthesia could enhance the sensitization of an individual to halothane and MAA adducts, thereby increasing their risk to cardiovascular disease.

Therefore, we hypothesize that halothane pre-treatment may potentiate the inflammatory response induced by the MAA-adduct as determined by TNF-α release. Thus, this rat-model study evaluates the effects of halothane pre-treatment in combination with an alcohol diet on *in vitro *HEC TNF-α release following stimulation with the MAA-adduct.

## Methods

### Chemicals and proteins

Bovine serum albumin (Alb) was purchased from CalBiochem (La Jolla, CA). Acetaldehyde (AA) was obtained from Aldrich Chemical Co. (Milwaukee, WI). Malondialdehyde (MDA) was obtained as the sodium salt (MDA~Na) by treatment of tetramethoxypropane (Aldrich Chemical Co.) with NaOH, according to the method of Kikugawa and Ido [14]. Lipopolysaccharide and Eschericahia coli 0111:B4 (LPS) was purchased from Sigma Chemical Co. (St. Louis, MO). Halothane was purchased from Halocarbon Laboratories (River Edge, NJ).

### Production of the malondialdehyde-acetaldehyde adduct

MAA-Alb was prepared as described by Tuma et al. [15] Briefly, Alb was modified with 1 mM malondialdehyde (MDA) and 1 mM acetaldehyde (AA) by incubating at 37 degrees for 72 hours. Following incubation, free and reversibly-bound MDA or AA was separated by exhaustive dialysis against a phosphate buffer for 24 hours at 4 degrees. Fluorescence measurements were obtained on post dialysis samples using a Perkin Elmer (Norwalk, CT) LS-5B spectrophotofluorometer attached to a Perkin Elmer GP-100 graphics printer as previously described [15]. Protein concentrations were measured as described by Bradford [16].

### Animal preparation

Male Wistar rats purchased from Charles River Laboratories (Willmington, MA) were maintained on a Purina rat chow diet, until they reached a weight of 140–150 grams, and were divided into three groups. These three groups were housed individually and acclimated to the Lieber-DeCarli liquid control diet from Dyets, Inc. (Bethlehem, PA) for 3 days [17]. The rats were paired by weight, one rat was given the ethanol-containing diet *ad libitum*, and the other rat was fed an isocaloric amount of the control liquid diet as determined by the pair-fed rat from the day before. Pair feeding was continued for 6 weeks. Finally, the ethanol-containing diet consisted of 18% of the total calories as protein, 35% as fat, and 36% as ethanol. In the control liquid diet, ethanol was replaced isocalorically with carbohydrates. The final group was given free access to standard laboratory chow and water.

For adduct immunization rats were injected once per week for 3 consecutive weeks beginning on day fourteen with one of the following protocols: (1) An injection of Alb only (25 μg/ml) subcutaneously plus an i.p. injection of an equal volume of sesame oil were given to control animals.; (2) halothane as a 21.5% solution in sesame oil at a dose of 10 mmol/kg intraperitoneally (i.p.);. (3) MAA-Alb (25 μg/ml) subcutaneously (s.c.); or (4) MAA-Alb and halothane combined in the previously mentioned doses. After one month (day twenty nine) on their respective diets, and 24 hours following the final injection of Alb, halothane (i.p.), MAA-Alb (s.c.), or MAA-Alb (s.c.) and halothane (i.p.) combined, the rats were sacrificed, and hearts removed for use in *in vitro *studies as described below. All animals were allowed free access to their food and/or water up to 1 hour before sacrifice. All procedures were approved by the animal subcommittee of the Omaha VA Medical Center, and are in accordance with the National Institutes of Health Guidelines on the Use of Laboratory Animals.

### Transaminase assay

Animals injected with the above ligands were bleed prior to sacrifice and serum transaminase enzymes determined using an (ALT/GPT and AST/GOT) assay kit purchased from Sigma Diagnostics (St. Louis, MO).

### Isolation and culture of heart endothelial cells (HECs)

Male Wistar rats were anesthetized intraperitoneal with phenobarbital (100 mg/kg) and the intact beating heart was immediately removed under sterile conditions. After mincing and dispase digestion, heart endothelial cells (HECs) were isolated and grown to confluency as previously described [9,18]. In brief, HECs were separated by centrifugation at 400 × g for 10 min followed by three washes with M199-F12 (GIBCO, Grand Island, NY) containing 10% fetal bovine serum (GIBCO). Cells collected were >90% HECs, verified by staining with mouse anti-rat RECA-1 (Harlan Bioproducts for Science, Indianapolis, IN) and mouse anti-Factor VIII-von Willebrand's Factor (Cedar Lane Laboratories Limited, Hornby, Ontario, Canada) [9]. Cells were seeded into 24 well tissue culture plates (Becton-Dickinson Labware, Franklin Lakes, NJ) containing fibronectin (20 μg/well) (Sigma Chemical Company, St. Louis, MO) and grown to confluency at 37°C for 48–72 hours.

### Percentage of cell necrosis determinations

The percentage of cell necrosis (death) of HECs during exposure to MAA-Alb was determined by an enzyme (lactic acid dehydrogenase, LDH) release assay of the HEC supernatant as described by Korzeniewski and Callewaert [19]. Briefly, following stimulation of HECs with 1,5,10, and 25 μg/ml MAA-Alb or media only (control) for 3 and 24 hours, the HECs were centrifuged (200 × g, 10 min) and a 100 μl aliquot of the HEC supernatant was transferred to the corresponding wells of flat-bottomed microtiter plates. Subsequently, 100 μl of a freshly prepared lactic acid dehydrogenase substrate mixture [5.4 × 10^-2 ^lactate (Acros Organics, New Jersey, USA), 6.6 × 10^-4 ^M 2p-iodophenyl-3p-nitrophenyl tetrazolium chloride (Acros), 2.8 × 10^-4 ^M phenazine methosulfate (Acros), and 1.3 × 10^-3^M nicotineamide nucleotide NAD in 0.2 M Tris buffer, pH 8.2 (Sigma)] was added to each well. The plates were incubated in the dark at room temperature for 10 min and the reaction stopped by the addition of 50 μl/well of a 1 M HCl solution. A microtiter plate reader (MR 7000, Dynatech Labs, Inc., Chantilly, VA) was used to monitor the resultant light absorbance at 490 nm while 630 nm was used as reference. LDH activity, expressed as change in absorbance/min, was calculated with Biolinx 2.21 software (Dynatech) on an IBM compatible computer. Percentage necrosis of the HECs was determined by the following formula: **% Necrosis = (E-S)/(M-S) × 100 **[19], where E is the optical density (OD) of the experimentally induced release of LDH activity from the HECs incubated in the presence of the various concentrations of MAA-Alb, S is the spontaneous release of LDH activity (OD) from HECs incubated with media only, and M is the maximal release of LDH activity (OD) determined by total HEC necrosis induced by exposure to 10% Triton X-100 (Fisher Scientific, Fair Lawn, NJ) [19].

### Endotoxin assay for LPS contamination

Prior to any stimulation all ligands, buffers, and media were tested for LPS content, which could influence the levels of background cytokine secretion. Samples were monitored for endotoxin using a Limulus Amebocyte Lysate assay from BioWhittaker (Walkersville, MD). Those samples contaminated with LPS at concentrations greater than 0.1 ng/ml were not utilized in these studies.

### MAA-Alb stimulation of HECs

HECs were washed on the day of the experiment with M199-F12 without serum and allowed to incubate for 1 hour to remove excess serum components. Following this incubation period, cells were stimulated with: 5 μg/ml Alb, MAA-Alb, LPS, and 10 μM Hexyl-MAA (a synthetic analog to the MAA-adduct) in serum free M199-F12 for 3 hours. Supernatant was collected and frozen at -70 degrees until assayed using a commercially available TNF-α ELISA kit.

### TNF-alpha ELISA

Quantification of TNF-α levels of the HEC supernatants was performed with a Factor-Test-X ™ rat TNF-α ELISA kit (Genzyme Diagnostics, Cambridge, MA), which employs a multiple antibody sandwich principle. The ELISA kit was developed, stopped and read at 450 nm on a MR7000 plate reader using BIOLINX ™ software. Final concentrations of TNF-α is expressed in pg/ml.

### Statistical analysis

All results are reported as means ± Standard Deviation (SD). Analysis of variance (ANOVA) was used to compare means between treatment groups. Dunnett's two-tailed *t*-test was used to determine if any pre-treatment was significantly different when compared to the unpretreated (control) group of similar diet and *in vitro *stimulant conditions. P values of 0.05 or less were regarded as statistically significance.

## Results

### Transaminase release

In an effort to determine liver damage from the administration of halothane and the MAA-adduct, serum from these animals were collected and assayed for the release of the serum transaminases, ALT and AST. Results indicated no difference between the animals injected with Alb, halothane, MAA-Alb, or halothane + MAA-Alb. There was a slight increase in ALT/AST levels in the ethanol-fed animals, yet these results were determined to be insignificant.

### Effects of increasing concentrations of MAA-Alb on *in vitro *HEC cell death

In order to determine what concentrations of MAA-Alb would result in cell death of HECs, cells were isolated from chow-fed rats and stimulated with increasing doses of the antigen. As shown in Table 1, HECs incubated with media alone, 1 and 5 μg/ml of MAA-Alb had little effect on % cell death after 3 and 24 hours of incubation. However, both 10 and 25 μg/ml of MAA-Alb had a significant increase in cell death over the control and lower concentrations of the same antigen. There was a statistical difference in the amount of cell death observed when HECs were exposed to10 μg/ml for 24 hours as compared to the 3 hour stimulation period. However, these differences were not observed with 25 μg/ml of MAA-Alb. For these reasons, 5 μg/ml of MAA-Alb was chosen as the optimum concentration for use in the remainder of the experiments in this manuscript.

**Table 1:** The percentage of cell death of heart endothelial cells (HECs) after stimulation with MAA Alb as determined by LDH release.

Time	10 μg/ml Alb	1 μg/ml MAA-Alb	5 μg/ml MAA-Alb	10 μg/ml MAA-Alb	25 μg/ml MAA-Alb
3 hours	1.8 ± 0.37	2.8 ± 0.55	2.6 ± 0.68	7.5 ± 0.70*	11.2 ± 0.58*
24 hours	2.6 ± 0.67	3.2 ± 0.58	3.0 ± 0.89	13.3 ± 0.53*	13.3 ± 0.71*

Results are expressed as means +/- SD for 6 determinations in each group. Values different from the media control are indicated (*) at P < 0.05.

### Effects of pre-treatment with halothane on TNF-α release by HECs

**Figure 1:**
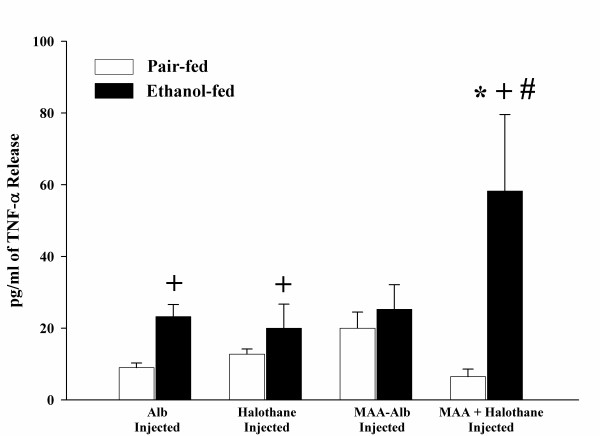
Alb-stimulated release of TNF-α by HECs isolated from pair and ethanol-fed rats immunized with Alb, halothane, MAA-Alb, or halothane + MAA-Alb. Results are expressed as means +/- SD for 5 experiments. Values different from Alb are indicated (*) at P < 0.01. Values different from the pair-fed control group are indicated (+) at P < 0.05. Values different from Halothane or MAA-Alb injected animals are indicated at (#) P < 0.001.

**Figure 2:**
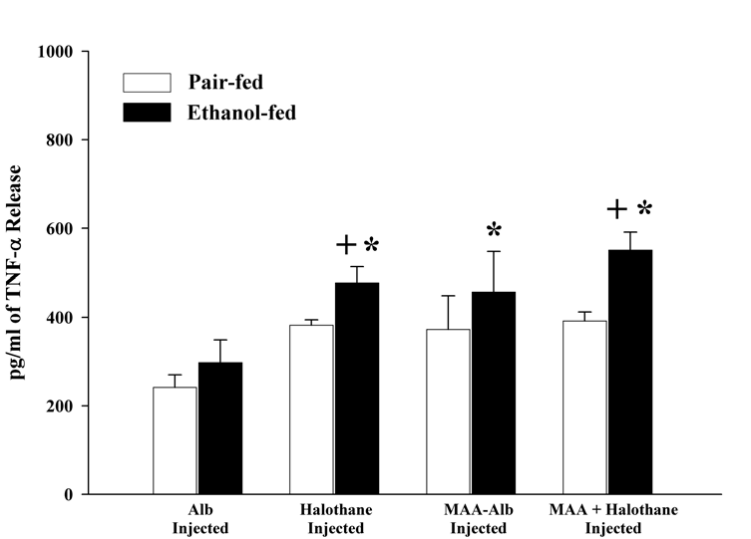
LPS-stimulated release of TNF-α by HECs isolated from pair and ethanol-fed rats immunized with Alb, halothane, MAA-Alb, or halothane + MAA-Alb. Results are expressed as means +/- SD for 5 experiments. Values different from Alb are indicated (*) at P < 0.05. Values different from the pair-fed control group are indicated (+) at P < 0.05.

In order to determine the effects of halothane pre-treatment on the release of TNF-α by HECs, the cells were isolated from pair and ethanol-fed rats that had been injected as previously described in the Materials and Methods with one of the following; Alb, halothane, MAA-Alb, or both halothane and MAA-Alb. The isolated HECs were stimulated *in vitro *with Alb, MAA-Alb, LPS, or Hexyl-MAA (a synthetic analog to MAA). As shown in Figure 1, HECs from ethanol-fed rats injected with MAA-Alb + halothane and stimulated with 5 μg/ml of Alb significantly (P < 0.01) increased the amount of TNF-α release when compared to animals injected with Alb. Increases in TNF-α release were observed in Alb, halothane, and MAA-Alb + halothane injected ethanol-fed animals in comparison to the pair-fed controls (P < 0.05). The most significant increase was demonstrated in the MAA + halothane injected ethanol rats when compared to the halothane or MAA-Alb injected animals (P < 0.001). As a positive control, LPS was used as the stimulating antigen, (Figure 2) and found to increase TNF-α secretion in the ethanol-fed animals as previously shown by others [20]. There was an increase over the Alb control in animals injected with halothane, MAA-Alb, and MAA + halothane (P < 0.05).

**Figure 3:**
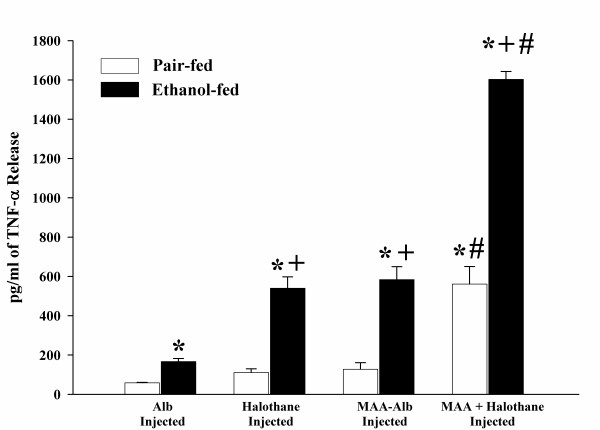
MAA-Alb-stimulated release of TNF-α by HECs isolated from pair and ethanol-fed rats immunized with Alb, halothane, MAA-Alb, or halothane + MAA-Alb. Results are expressed as means +/- SD for 5 experiments. Values different from the Alb injected group are indicated at (*) P < 0.001. Values different from pair-fed control group are indicated at (+) P < 0.001. Values different from Halothane or MAA-Alb injected animals are indicated at (#) P < 0.001.

**Figure 4:**
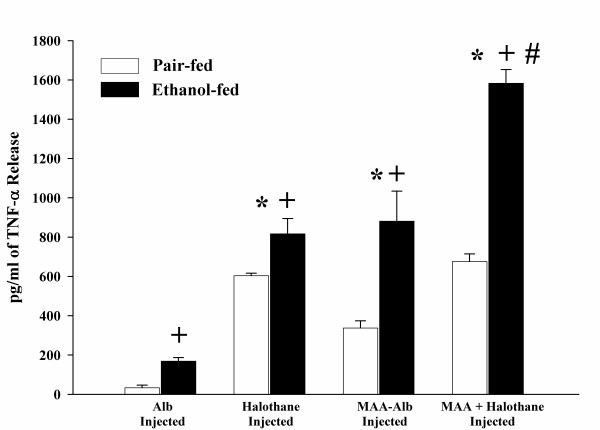
Hexyl-MAA-Alb-stimulated release of TNF-α by HECs isolated from pair and ethanol-fed rats immunized with Alb, halothane, MAA-Alb, or halothane + MAA-Alb. Results are expressed as means +/- SD for 6 experiments. Values different from the Alb injected group are indicated at (*) P < 0.001. Values different from pair-fed control group are indicated at (+) P < 0.001. Values different from Halothane or MAA-Alb injected animals are indicated at (#) P < 0.001.

Pair and ethanol-fed rats injected with the above antigens increased TNF-α release 3 fold in response to stimulation with MAA-Alb in comparison to the Alb or LPS stimulated HEC. As shown in Figure 3, increases in TNF-α release was demonstrated in the halothane, MAA-Alb, and halothane + MAA-Alb rat HECs after stimulation with MAA-Alb as compared to the Alb injected rat HECs (P < 0.001). When comparing the four groups of rats, TNF-α release was increased in HECs from ethanol-fed rats over the pair-fed controls (P < 0.001). Ethanol-fed rats injected with halothane or MAA-Alb had similar effects on HEC secretion of TNF-α, while MAA-Alb + halothane together had a 3 fold increase over halothane or MAA-Alb alone (P < 0.001). This synergistic response was also observed in the pair-fed controls for the MAA-Alb + halothane injected rat HECs. As witnessed in Figure 4, Hexyl-MAA (the synthetic analog of MAA) stimulated HECs similar to that of MAA-Alb. Increases in TNF-α secretion is witnessed in the ethanol-fed rats for all the experimental conditions (P < 0.001). There is a 2–3 fold increase in groups injected with halothane, MAA-Alb, or halothane + MAA-Alb over the Alb injected control (P < 0.001). The most significant increase is the 2 fold increase in the MAA-Alb + halothane injected ethanol-fed rats (P < 0.001). These data show an additive effect using the combination of both antigens.

## Discussion

Tumor necrosis factor-alpha (TNF-α), the most proximal pro-inflammatory cytokine mediator released following LPS (endotoxin) stimulation, induces an inflammatory response through several mechanisms, including increased neutrophil-endothelial cell adherence, increased endogenous nitric oxide production [21], and the stimulation of the production and release of other pro-inflammatory cytokines, including interleukin-8 (IL-8) [22]. Recently, the role of TNF-α in the process of apoptosis (cell death) has been demonstrated [23]. For example, while apoptosis occurs naturally in the liver at a low and controlled rate, an increased rate of apoptosis is observed following increased cellular TNF-α concentration. The direct correlation between TNF-α concentrations and the rate of apoptosis has been described in several types of liver diseases characterized by cell necrosis and death [24]. Apoptosis has also been demonstrated in heart endothelial cells in response to TNF-α and Interleukin-18 [25,26]. Cell death experiments demonstrate that the MAA-adduct caused cell death only at concentrations of 10 μg/ml or greater (Table 1). Since TNF-α may cause cell death, [24] the significant increase in the percentage of HEC cell death following stimulation with 10 and 25 μg/ml of MAA-Alb may be due to the direct toxicity of the MAA-adduct or indirectly due to MAA-adduct-induced TNF-α release. Further studies will be required to clarify this issue. Ohki *et al *[27] reported, consistent with the results of our study, that ethanol fed rats demonstrate greater TNF-α release when exposed to LPS, with the increased TNF-α release following ethanol feeding caused increased neutrophil-endothelial adherence. Other investigators have similarly demonstrated increased neutrophil-endothelial adherence induced by ethanol, suggesting ethanol ingestion induces an inflammatory injury [28]. Since the MAA-adduct is a primary metabolic end product of ethanol metabolism, our demonstration that this adduct induces TNF-α release and that alcohol feeding potentiates endotoxin-induced TNF-α release is consistent with the understanding that alcohol adduct induced TNF-α release may play a significant role in alcohol induced solid organ injury [20].

MDA has been detected in guinea pig heart tissue following exposure to halothane [29]. Also interesting is that circulating antibodies to cardiac protein-acetaldehyde adducts have been found in alcoholic heart muscle disease [30]. If AA from alcohol metabolism and MDA from halothane are present in heart tissue, the possibility of MAA-adduct formation is likely. The majority of adducts formed when acetaldehyde reacts with proteins for short time periods are unstable AA-protein adducts. With time, unstable AA-adducts stabilize and can form an irreversible adduct. This irreversible, stable adduct has been demonstrated to be the MAA-adduct [15]. These MAA-adducts have also been found in atherosclerotic human aortic heart tissue [9], providing a mechanism of heart tissue damage. Heavy alcohol consumption can accelerate human atherosclerotic heart disease [31], making MAA-adducts a possible candidate for this process.

Increased TNF-α levels have similarly been implicated in other disease states, like end-stage heart disease and intractable heart failure, by the promotion of monocyte dysfunction and death [32]. Since TFA-adduct production is induced with a single dose of halothane and persists in measurable concentrations in rat heart for greater than 90 hours (but less than 10 days), [8,33] the possibility for cross-reaction with MAA-adducts is plausible. These experiments demonstrated that halothane and the MAA-adduct-induced HEC TNF-α release in a synergistic manner. In support of this data, Trudell *et al *[3] demonstrated that antibodies raised against AA-adducts and TFA-adducts cross-react, suggesting that the immunologic properties of both adducts may be similar. This data suggests that similar immunologic mechanisms may be shared by both halothane hepatitis and ethanol-induced liver injury. The data reported in this study demonstrates that halothane pre-treatment will potentiate the MAA-adduct induced TNF-α release *in vitro *of HECs. This gives support to the Trudell *et al *[3] demonstration that the AA-adducts and TFA-adducts induce organ injury by the release of chemoattractants during the metabolism of ethanol and halothane, resulting in the recruitment of inflammatory cells as the initial step in the initiation of organ injury. TNF-α production and release is a primary biologic mechanism in inflammatory cell recruitment since TNF-α induces the production of IL-8 [34]. Interleukin-8 is the primary cytokine responsible for the promotion of inflammatory cell (neutrophils and monocytes) chemotaxis (migration) and recruitment toward an inflammatory site [34]. Further studies need to be done in order to prove that the TFA-adduct and MAA-adduct cross-react.

## Conclusion

In conclusion, the current study demonstrates that halothane or MAA-Alb pre-treatment potentiates the HEC TNF-α release following MAA-adduct stimulation of ethanol-fed rats when compared to control pair-fed rats. This suggests that the TFA-adduct resulting from the metabolism of halothane increases the inflammatory response, as measured by TNF-α release, following LPS and alcohol (MAA-Alb) adduct stimulation, and that this TNF-α release may contribute to post-halothane exposure solid organ injury. The data also suggest that solid organ injury following halothane administration may be enhanced by prior ethanol consumption. This could help explain the increased risk of cardiovascular disease following excessive alcohol consumption. Finally, these results demonstrate that the combination of ethanol consumption and halothane exposure may enhance the possibility of the development of the sepsis syndrome, since increased systemic concentrations of TNF-α induced by endotoxin is a primary cause of that clinical condition.

## Competing interests

The author(s) declare that they have no competing interests.

## Authors' contributions

GMT was the originator of the concept, wrote the article, and participated in the design, coordination, and implementation of the study. GEH participated in the design, performed technical work, and participated in the writing. JAP participated in the design and performed technical work. TLF participated in the writing of the manuscript. DJT participated in the design of the study. MJD participated in the drafting of the manuscript and performed technical work. LWK participated in the design of the study and coordinated the experiments. All the authors approved the final draft of this manuscript.

## Pre-publication history

The pre-publication history for this paper can be accessed here:


